# Specifying the Efficacy of Digital Therapeutic Tools for Depression and Anxiety: Retrospective, 2-Cohort, Real-World Analysis

**DOI:** 10.2196/47350

**Published:** 2023-09-22

**Authors:** Yifat Fundoiano-Hershcovitz, Inbar Breuer Asher, Marilyn D Ritholz, Eitan Feniger, Omar Manejwala, Pavel Goldstein

**Affiliations:** 1 Dario Health Caesarea Israel; 2 Joslin Diabetes Center Harvard Medical School Boston, MA United States; 3 Integrative Pain Laboratory (iPainLab), School of Public Health University of Haifa Haifa Israel

**Keywords:** mental health, depression, anxiety, digital health, coaching, behavioral health, breathing exercises, behavioral change, digital health intervention, cognitive behavior therapy, health outcome, health app, intervention, cohort, retrospective

## Abstract

**Background:**

Depression and anxiety are the main sources of work and social disabilities as well as health-related problems around the world. Digital therapeutic solutions using cognitive behavioral therapy have demonstrated efficacy in depression and anxiety. A common goal of digital health apps is to increase user digital engagement to improve outcomes. However, there is a limited understanding of the association between digital platform components and clinical outcomes.

**Objective:**

The aim of the study is to investigate the contribution of specific digital engagement tools to mental health conditions. We hypothesized that participation in coaching sessions and breathing exercises would be associated with a reduction in depression and anxiety.

**Methods:**

Depression and general anxiety symptoms were evaluated in real-world data cohorts using the digital health platform for digital intervention and monitoring change. This retrospective real-world analysis of users on a mobile platform–based treatment followed two cohorts of people: (1) users who started with moderate levels of depression and completed at least 2 depression assessments (n=519) and (2) users who started with moderate levels of anxiety and completed at least 2 anxiety assessments (n=474). Levels of depression (Patient Health Questionnaire-9) and anxiety (Generalized Anxiety Disorder-7) were tracked throughout the first 16 weeks. A piecewise mixed-effects model was applied to model the trajectories of the Patient Health Questionnaire-9 and the Generalized Anxiety Disorder-7 mean scores in 2 segments (1-6 weeks and 7-16 weeks). Finally, simple slope analysis was used for the interpretation of the interactions probing the moderators: coaching sessions and breathing exercises in both depression and anxiety cohorts.

**Results:**

Analysis revealed a significant decrease in depression symptoms (*β*=–.37, 95% CI –0.46 to 0.28; *P*≤.001) during the period of weeks 1-6 of app use, which was maintained during the period of 7-16 weeks. Coach interaction significantly moderated the reduction in depression symptoms during the period of weeks 1-6 (*β*=–.03, 95% CI –0.05 to –0.001; *P*=.02). A significant decrease in anxiety symptoms (*β*=–.41, 95% CI –0.50 to –0.33; *P*≤.001) was revealed during the period of 1-6 weeks, which was maintained during the period of 7-16 weeks. Breathing exercises significantly moderated the reduction in anxiety symptoms during the period of 1-6 weeks (*β*=–.07, 95% CI –0.14 to –0.01; *P*=.04).

**Conclusions:**

This study demonstrated general improvement followed by a period of stability of depression and anxiety symptoms associated with cognitive behavioral therapy–based digital intervention. Interestingly, engagement with a coaching session but not a breathing exercise was associated with a reduction in depression symptoms. Moreover, breathing exercise but not engagement with a coaching session was associated with a reduction of anxiety symptoms. These findings emphasize the importance of using a personalized approach to behavioral health during digital health interventions.

## Introduction

Depression and anxiety are the main sources of social and work disabilities as well as health-related problems around the world and the prevalence of these common mental health disorders is increasing [1,2]. Furthermore, the COVID-19 pandemic has reinforced the need to strengthen mental health treatment worldwide by offering more robust services during times of more restricted personal movement [2]. The burden of depression and anxiety is elevated across the entire lifespan, with no evidence of global reduction since 1990 [3]. For example, major depression places fourth on the list of disorders with the highest burden of disease and is expected to be on the top of the list in high-income countries by 2030 [4].

Depressive and anxiety disorders have a major impact on managing daily life and interfere with work and productivity [5]. In this study, we analyzed the follow-up data of 2 mental health–related cohorts during the digital intervention. We investigated the nonlinear behavior path for digital health intervention for mental health, suggesting that coaching and breathing exercises are exclusively boosting the improvement of depression and anxiety symptoms correspondently.

These mental health disorders are also major public health problems among working-age adults [6]. Several studies have shown the immense economic challenge these disorders pose to communities and society at large as a result of health and social care expenditures [7,8]. Efforts to reduce work impairment caused by depression are crucial [9].

There are various psychological interventions for anxiety disorders and evidence supporting their effectiveness in the past decades [10-12]. Previous research has shown that depressed primary care patients preferred psychological treatments over psychiatric drug treatment and research found that patients who did not receive their preferred form of treatment evidenced lower working alliance scores [4,6,13,14].

The COVID-19 pandemic intensified depression and anxiety symptoms and people are experiencing difficulties in accessing appropriate psychological treatment [15]. Digital mental health services have the potential to expand the accessibility of mental health care [15]. The Unified Protocol for Transdiagnostic Treatment Anxiety and Depressive Disorders (UP) is a well-regarded framework in the field of mental health. While it was initially developed as a face-to-face therapy, its principles and adaptability make it a promising framework in the context of digital health interventions. UP is based on Cognitive Behavioral Therapy and was designed to be used for the treatment of a broad range of emotional disorders and applied for individuals diagnosed with anxiety disorders, depression, and related disorders supported by clinical evidence [16-21]. A common language and a specific set of treatment principles are used in transdiagnostic protocols like UP in order to target various clinical or comorbid conditions typically, anxiety and depression [22]*.* The current interest in digital therapeutics for mental health is highly driven by the need to increase access to mental health services and presents new treatment opportunities [23]. The UP approach allows for more efficient and effective delivery of therapeutic tools by addressing shared mechanisms rather than focusing on disorder-specific protocols. In a digital health setting, this flexibility can cater to a broader range of users with different emotional disorders. The intervention based on UP can be delivered directly to individuals with high reliability and leveraging technology platforms such as smartphones, empowers individuals to self-manage mental health conditions [24,25]. Digital therapeutics technology presents an opportunity to transform mobile phones into devices that could provide global, cost-effective, and evidence-based mental health services on demand and in real-time [26]. There has been an increase in recent years in the use of mobile health platforms to intervene in the management of anxiety and depression and these 2 disorders have the highest prevalence among mental health conditions [27]. Digital interventions use features such as module-based sessions and teach coping and management techniques, problem-solving therapy, and psychoeducation [27]. Digital health services for mental health provide extensive access and increase cost-effectiveness compared with traditional face-to-face interventions [21,28,29]. Technology may include incorporating automated tailored messages; reporting thoughts, feelings, or behaviors; recommending exercises; provision of mental health information; and real-time engagement or activities explicitly linked to specific reported mood problems [30]. Empirical evidence has shown that engagement with a digital mental health intervention is associated with therapeutic benefits [25] and was even seen as similarly efficacious as traditional face-to-face treatments [21,29].

Coaching has been proposed to facilitate improvements in depression in a personal capacity targeting broad life challenges and empowering users to formulate their own solutions [31]. Prior studies have shown that digital mental health interventions may be highly effective for mental health disorders such as depression when incorporating in-person elements [32]. Supporting coaching was shown as an effective approach to creating positive change, facilitating goal attainment using problem-solving, and enhancing self-efficacy [31]. It was demonstrated that participating in a digital video coaching session was associated with lower depression symptoms in the immediate week [33].

Participating in digital coaching sessions was found to contribute to durable decreases in depression [33-36]. Further research is required to better understand the specific effect of coaching on mobile mental health, specifically in depression [32,37] and the temporal dynamics of the digital therapeutic approaches should be investigated [38]. To further understand the value and specific therapeutic contribution of UP-based tools, we tested the effect of coaching on depression and anxiety outcomes, suggesting the supportive role of coaching for users with depression symptoms.

On the other hand, breathing techniques are mostly used as primary supplemental treatments for stress and anxiety [39]. Previous studies have demonstrated a reduction in stress levels after 12 weeks of breathing exercises linked to an increased feeling of well-being and a reduction in anxiety [40]. Recent research has revealed intriguing connections between anxiety and autonomic, respiratory, and cardiac activity, hinting at potential underlying mechanisms involving cellular membrane potential changes in response to physiological alterations induced by various emotional states [39,41,42]. There are specific characteristics of sympathetic or parasympathetic activity and respiration that are related to certain emotional states such as anxiety or happiness. Emotional states such as anxiety or fear tend to be associated with increased sympathetic activity and may lead to rapid, shallow breathing. Nonetheless, emotions like happiness and relaxation are associated with increased parasympathetic activity. This can lead to slower, deeper breathing, which has a calming effect on the body [39,43,44]. Principally, deep breathing increases oxygenation and inhibits excitatory activity and widespread depolarization leading to emotional calming [45,46].

Previous studies have shown the efficacy of using deep breathing techniques as a part of stress and anxiety management programs [47,48]. With the widespread everyday use of smartphones and regarding the benefits of digital mental health programs [47], such as accessibility issues, cost, and convenience, further research is required to understand the specific efficacy and temporal dynamic of digitally delivered breathing exercises component on anxiety symptoms.

Evidence-based workplace interventions may be a key component to prevent the development of and decrease symptoms of depression or anxiety among adults. Workplace interventions may also help establish a set of therapeutic tools for depressive and anxious conditions.

Although more than 10,000 mental health apps are available today, very few (around 4%) have demonstrated clinical efficacy [49-51]. To date, there have been very few large-scale studies examining the effectiveness of digital therapeutic interventions for emotional disorders delivered in real-world settings. The lack of effectiveness across specific platforms may be attributable to a limited available evidence base for many of the interventions [52-54]. There also is limited quantitative literature on the associations between the program components and clinical outcomes [55]. Most of the interventions developed were examined in strictly controlled study environments with challenges such as lower participant engagement that may reflect the narrow interests of a small, biased group of researchers and do not translate into a “real-world” environment [52,56]. Clinical significance refers to the practical importance of the changes observed in individuals’ mental health symptoms or overall well-being. In general, modeling the use of clinical significance as an outcome in the digital health real-world data can contribute to the advancement of mental health interventions. Further evidence is required to better understand the effectiveness of digital health tools as well as their moderating factors [6,25,35].

Importantly, the process of mental health healing using digital therapeutic tools is not linear [57,58] and should be appropriately modeled. Moreover, the efficacy of digital therapeutics for mental health conditions shows strong heterogeneity [53,59], while users’ efforts and training may boost healing.

Many evidence-based therapies for mental health are typically structured as time-limited interventions spanning several weeks. A 16-week time frame aligns with the duration of such interventions, allowing for comprehensive modeling of the treatment process and outcomes. The UP-based interventions have previously demonstrated mental disorder symptom reduction following 16 weeks of treatment [60]. Digital tools and coaching are expected to have a measurable impact on users over an 8-12 week window [61-63]; there is additional evidence for a change in behavioral activation correlated with a 6-week self-help program [37]. A 16-week time frame provides an opportunity to assess the sustainability of treatment effects beyond the immediate period. By evaluating outcomes at multiple time points within the 16-week period, it becomes possible to examine if the observed changes in mental health symptoms are maintained over time.

We suggested that during the initial period of using a behavioral health platform, experiencing coaching and breathing exercises would be associated with reduced depression and anxiety levels correspondently. By modeling the 2-stage trajectory process, we expected to show the improvement in depression and anxiety symptoms to persist during the initial 6-8 weeks of the intervention, followed by symptom maintenance until week 16. We hypothesized that (1) coach interaction will moderate the depression symptom recovery and (2) breathing exercises will moderate anxiety symptom recovery. We also explored the moderating effect of app session completion and watched video sessions on depression and anxiety symptom improvement.

## Methods

### Platform

The Dario behavioral health platform is essentially a modular, transdiagnostic tool delivering emotion-focused support designed to be applicable to a range of mental health conditions. Members generally have access to the Dario Health behavioral health platform as part of their employee or health plan benefits. A clinically based screening tool assesses each person’s needs and guides users to the most efficient support. The platform was built using the UP framework for depression, anxiety, anger, stress, and substance use and can be used as a self-guided intervention or with the help of a certified coach. The UP is a recently developed transdiagnostic treatment that was designed to be used for the treatment of anxiety and depressive disorders [64]. Members using the digital program complete modules based on the UP framework. Coaches assist where members have challenges focusing on accountability and goal setting.

The structure of each program is composed of app sessions including conceptual videos, textual skills, breathing exercises, and monitoring progress tools. This study focuses on tracking cohorts of users with depression and anxiety scores measured by their responses to Patient Health Questionnaire-9 (PHQ-9) and Generalized Anxiety Disorder-7 (GAD-7) questionnaires.

In the depression program, a broad overview of depression is provided, and users receive a variety of literature-supported reasons why depression exists, statistics to help normalize its presence, and a framework for why depressive symptoms may be different for everyone. Subsequent program content introduces empirically supported techniques for depression, including activity scheduling, behavioral momentum, mindfulness, and relaxation-based strategies and coaching. Later sessions introduce the role of automatic thoughts, strategies for managing them, values-based interventions, and relapse prevention skills.

The anxiety program begins with psychoeducation about generalized anxiety, how it differs from “normal” anxiety, as well as how it presents itself physically, mentally, and emotionally. The latter portion of the program introduces coping strategies, including the relationship between thoughts, feelings, and behaviors, and ways to influence these interconnected variables in a positive manner. These include supported skills in cognitive strategies to challenge and alter anxiety-promoting thoughts. In addition, during these skills-based sessions, various mindfulness and relaxation skills and coaching are introduced to assist with reducing anxiety.

Coaching and breathing exercises are introduced as a support component in the digital health platform for users with depression and anxiety symptoms and can be a valuable addition to their overall care. Coaching can provide personalized guidance, motivation, and support to users as they navigate their mental health journey.

### Measures

Depressive symptoms were tracked over 16 weeks using the PHQ-9, a 9-item self-reported, in-app delivered questionnaire designed to evaluate the presence of depressive symptoms. Each of the 9 items, asking for each of the DSM-IV (Diagnostic and Statistical Manual of Mental Disorders, 4th Edition) diagnostic criteria, is scored from 0 (not at all) to 3 (nearly every day). The sum score ranges from 0 (absence of depressive symptoms) to 27 (severe depressive symptoms). Scores between 0 and 4 signify the absence of a depressive disorder, scores from 5 and 9 indicate a mild level of depressive symptoms, scores from 10 and 14 indicate a moderate level of depressive symptoms, scores between 15 and 19 indicate moderately severe major depression, and a score of 20 and above indicate a severe level of depression [65,66]. The PHQ-9 has demonstrated good internal reliability with a Cronbach α of .83 [67]. Anxiety symptoms were tracked over 16 weeks using the GAD-7, a 7-item self-reported anxiety questionnaire designed to assess the patient’s health status. Each of the items is scored from 0 (not at all) to 3 (nearly every day). The sum score ranges from 0 (absence of anxiety symptoms) to 21 (severe anxiety symptoms). Scores between 0 and 4 signify the absence of anxiety symptoms, scores between 5 and 9 indicate a mild level of anxiety, scores between 10 and 14 indicate a moderate level of anxiety and a score of 15 and above indicates a severe level of anxiety [68]. The internal reliability of the GAD-7 is high with a Cronbach α of .86 [69].

Independent variables included in the analysis as potential moderating factors were self-use sessions, breathing exercises, and coach interactions, operationalized as the number of times a user experienced an activity each week, and available demographic variables such as gender. All data were transferred and stored in compliance with Health Insurance Portability and Accountability Act (HIPAA) requirements, using MongoDB or BigQuery database services. All data were anonymized before extraction for this study.

### Users

A retrospective data evaluation study was performed on the Dario database.

The depression cohort consisted of a group of 519 platform users who used the Dario Behavioral Health platform between 2019 and 2021. The sample included 389 (75%) women, 118 (23%) men, and 12 (2%) others. The age distribution was as follows: <35 (47%), 36-45 (26%), and >45 (27%) years. The anxiety cohort consisted of a group of 474 platform users who used the Dario Behavioral Health platform between 2019 and 2021. The sample included 353 (75%) women, 110 (23%) men, and 11 (2%) others. The age distribution was as follows: <35 (47%), 36-45 (25%), and >45 (28%) years.

### Study Design

We applied a retrospective cohort study design to follow depression and anxiety symptoms over time. We used a database containing previously recorded follow-up data from the Dario Health users. The inclusion criteria for the depression cohort included users who started at a moderate and above level of depression (PHQ-9 mean score of >9) and those who completed at least 2 PHQ-9 assessments. The inclusion criteria for the anxiety cohort included users who started at a moderate and above level of anxiety (GAD-7 score of >9) and those who completed at least two GAD-7 assessments.

### Ethical Considerations

This study is based on an analysis of an existing database already collected in the digital platform. All data were anonymized before extraction for this study. Ethical and Independent Review Services [70], a professional review board, issued the institutional review board exemption for this study (21235-01#).

### Statistical Analysis

Previous studies consistently demonstrated a 2-stage dynamic of clinical outcomes associated with digital interventions: an initial improvement is followed by stabilization of the outcome [57,58]. A classical linear longitudinal model assumes a single-slope growth pattern for changes in an outcome variable across time. In contrast, piecewise‐based mixed‐effects models allow flexibility in the modeling of variable change trajectories across time [71]. The model is based on a set of linear slopes, shaping curvilinear behavior of the outcome over time.

Following visualization of the distribution of the depression and anxiety estimates time, the piecewise cutoff point for the model slopes was chosen at 6 weeks of platform use, assuming a change in the time-related depression severity level trajectory after 6 weeks [61-63]. Such behavior is in line with our previous research on digital follow-up measures in chronic conditions management [72-74].

Here, a piecewise mixed-effects model was conducted to model the trajectories of the PHQ-9 and the GAD-7 mean scores in 2 segments (1-6 weeks and 7-16 weeks) to allow the data to exhibit different linear trends over different time regions.

Next, we tested whether interactions with the coach moderate 2 piecewise time trajectories in depression and anxiety (1-6, 7-16 weeks). In addition, breathing exercises use was tested as a moderator of depression and anxiety fluctuations over both time periods. Simple slope analysis was used for the interpretation of the interactions probing the moderators at 1 SD below (low use) and above (high use) the average. The same statistical framework was applied to test the moderating effect of the number of app sessions completed and the number of watched video sessions.

## Results

### Study 1: Depression Symptoms Analysis

A total of 63% of the users showed an overall improvement in their depression levels over the study period. Moreover, 190 out of 519 (37%) of the users showed a reduction of ≥5 points (generally considered clinically significant [75]) in their depression levels over this period. The dropout rate was 25.2%.

**Figure 1:**
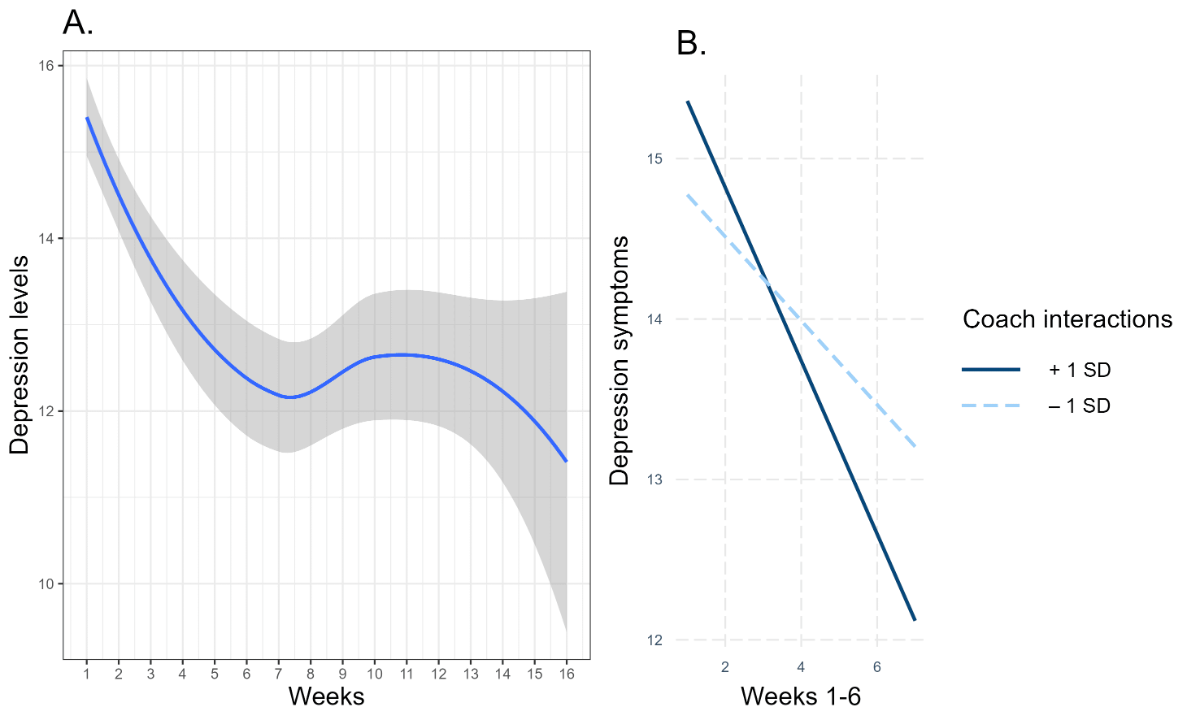
Depression symptom fluctuation over 16 weeks of app use. (A) The blue line presents less smoothing of depression symptoms over time. The gray area around the line represents 95% CI. (B) Simple slope analysis of the depression time trajectories during weeks 1-6 weeks of app use moderated by the interaction with a coach.

Figure 1A demonstrates the time-related fluctuation of depression symptoms. Piecewise mixed model analysis revealed a significant decrease in depression symptoms (*β*=–.37, 95% CI –0.46 to 0.28; *P*≤.001) during the period of weeks 1-6 of the platform use. There were no significant time-related trends in depression during the period from 7 to 16 weeks (*β*=–.09, 95% CI –0.21 to 0.02; *P*=.11).

**Figure 2:**
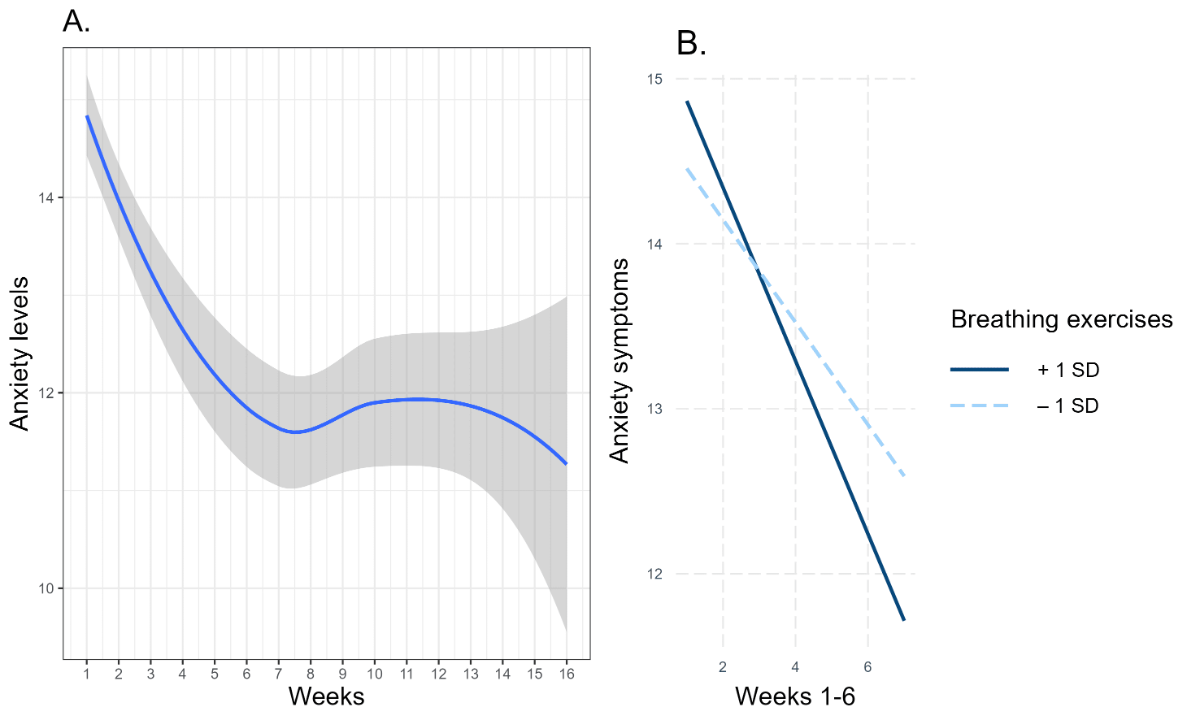
Anxiety symptom fluctuations over 16 weeks of app use. (A) The blue line presents less smoothing of anxiety symptoms over time. The gray area around the line represents 95% CI. (B) Simple slope analysis of the anxiety time trajectories during weeks 1-6 of app use moderated by breathing exercises.

Coach interaction significantly moderated the reduction in depression symptoms during the period of weeks 1-6 (*β*=–.03, 95% CI –0.05 to –0.001; *P*=.02). Users with increased coach interactions (+1 SD) demonstrated a stronger reduction in depression (*β*=–.59, 95% CI –0.81 to –0.36; *P*≤.001) comparing to the users with low coach interactions level (–1 SD) that was also significant (*β*=–.23, 95% CI –0.46 to 0.00; *P*=.047) (Figure 2). Coach interactions did not moderate the time trajectory of depression symptoms during the period of 7-16 weeks (*β*=.00, 95% CI –0.03 to 0.04; *P*=.75).

Figure 1B presents low and high levels of interaction with a coach showing a significant reduction in depression symptoms but the reduction is stronger for the users with higher levels of interactions.

Breathing exercise did not significantly moderate a reduction in depression symptoms in both periods of time (weeks 1-6: *β*=–.05, 95% CI –0.12 to 0.02; *P*=.16 and weeks 7-16: *β*=–.02, 95% CI –0.12 to 0.09; *P*=.76).

The number of the completed sessions did not moderate the time trajectory of depression symptoms during the period of weeks 1-6 (*β*=–.01, 95% CI –0.06 to 0.03; *P*=.55) and 7-16 (*β*=–.03, 95% CI –0.13 to 0.06; *P*=.48). In addition, the number of watched videos did not moderate the time trajectory of depression symptoms during the period of 1-6 (*β*=–.02, 95% CI –0.06 to 0.01; *P*=.21) and 7-16 week (*β*=–.02, 95% CI –0.05 to 0.02; *P*=.37).

### Study 2: Anxiety Symptoms Analysis

A total of 63% of the users showed an overall improvement in their anxiety levels over the study period. Moreover, 159 out of 474 (34%) of the users showed a reduction of ≥5 points (4 points is generally considered clinically significant [76]) in their anxiety levels. The dropout rate was 28.4%.

Figure 2A demonstrates the time-related fluctuation of anxiety symptoms. Piecewise mixed model analysis revealed a significant decrease in anxiety symptoms (*β*=–.41, 95% CI –0.50 to –0.33; *P*≤.001) during the period of 1-6 weeks of app use. There were no significant time-related trends in anxiety during the period of 7-16 weeks (*β*=–.09, 95% CI –0.19 to 0.02; *P*=.12).

Breathing exercise significantly moderated the reduction in anxiety symptoms during the period of 1-6 weeks (*β*=–.07, 95% CI –0.14 to –0.01; *P*=.04). Users who frequently used breathing exercises (+1 SD) demonstrated significant reductions in anxiety (*β*=–.53, 95% CI –0.66 to –0.39; *P*<.001) comparing to users who were less engaged with breathing exercises (–1 SD) that was also significant (*β*=–.31, 95% CI –0.44 to –0.18; *P*<.001). The time-related anxiety symptom trajectory during the period of 7-16 weeks was not moderated by breathing exercises (*β*=–.04, 95% CI –0.14 to 0.05; *P*=.34).

Figure 2B demonstrates the moderating role of breathing exercises in the anxiety symptoms fluctuation: users with high versus low use of breathing exercises showed a stronger reduction in anxiety symptoms. However, the reduction of anxiety symptoms over time was significant in both groups of users.

Coach interaction did not significantly moderate a reduction in anxiety symptoms in both periods of time (weeks 1-6: *β*=–.03, 95% CI –0.05 to 0.03; *P*=.68; weeks 7-16: *β*=–.01, 95% CI –0.05 to 0.04; *P*=.72).

The number of completed sessions did not moderate the time trajectory of anxiety symptoms during the period of 1-6 (*β*=–.02, 95% CI –0.07 to 0.04; *P*=.55) and 7-16 weeks (*β*=–.12, 95% CI –0.24 to 0.00; *P*=.057). In addition, the number of watched videos did not moderate the time trajectory of anxiety symptoms during the period of 1-6 (*β*=–.01, 95% CI –0.05 to 0.02; *P*=.37) and 7-16 weeks (*β*=.01, 95% CI –0.04 to 0.03; *P*=.68).

## Discussion

### Principal Results

This study tested the effect of a digital therapeutic product on depression and anxiety symptoms over time, specifying therapeutic instruments for mental health conditions. Piecewise mixed model analysis indicated a significant decrease in depression symptoms during the period of the first 6 weeks of the product use and maintained during the period from 7 to 16 weeks with no significant time-related trends. Exclusively coach interaction moderated the reduction in depression symptoms during the period of weeks 1-6. Our findings also revealed a significant decrease in anxiety symptoms during the period of 1-6 weeks. During the period of 7-16 weeks, the reduced level of anxiety was maintained, and there were no significant time-related trends. As expected, breathing exercises moderated the reduction in anxiety symptoms during the period of 1-6 weeks however did not moderate depression symptoms improvement. App sessions completion and watched video sessions did not moderate the reduction of either the depression symptoms or anxiety symptoms.

This real-world analysis presents new evidence regarding the dynamic efficacy of digital therapeutic solutions for people with depression or anxiety symptoms. These findings support the hypothesis of nonlinear depression and anxiety symptom recovery via digital therapeutic intervention [57,59]. Nonlinear effect of digital therapeutics has previously been demonstrated in studies of chronic condition management such as glucose control in diabetes [72], and also in fluctuations of blood pressure levels over time [73] and in pain levels and posture perception [74].

In mental health and psychotherapy, a period of critical fluctuation can be used to identify points of system transition, moving from stabilization to sustainability [77]. The point of this transition that was identified in our study was after 6 weeks in both cohorts; depression and anxiety symptoms were stabilized and sustained.

To explore the relationship between specific digital tools and improvement in mental health symptoms, we examined the effect of coach interactions via a digital platform. We hypothesized that coach interactions would moderate the reduction in depression symptoms. Health coaching may drive a change via motivational interviewing to create specific goals, or through mood improvement that enables the stabilization of mood swing experiences or patient-driven interactions based on social factors and life contexts [78,79] that are usually targeted in the depression interventions. The coach interaction component was previously demonstrated as a factor simulating an in-person experience and helped achieve positive outcomes and behavioral change in various conditions such as weight loss and diabetes [78,80].

The interpersonal relationship has been found to be the common factor in psychotherapeutic interventions [81]. Thus, coaching may offer the needed interpersonal support in the digital journey. It also can continue supporting the user experiencing depression in focusing on goals and challenges, as well as acting as assistance to the care plan. A coach may help users increase self-esteem and therefore, users may cease to see themselves as a failure and may deal with rejection and disapproval effectively, which may contribute to reduced depression symptoms.

Previous studies have shown that those who had a low level of interaction with a coach were consistently less likely to improve clinical outcomes than those with a high level in the follow-up period [62]. Moreover, research has demonstrated that the active use of digital interventions leads to behavior change related to better depression outcomes [35]. The clinical improvement associated with the moderators of treatment outcomes will assist in developing better digital health interventions for depression [35].

Our findings provide insight into the nonlinear nature of depression symptom reduction during the first 16 weeks of a digital behavioral health program by showing the direct association with coaching during the improvement stage in the first 6 weeks and emphasizing the key role of digital coaching in depression symptom reduction. In the next 7-16 weeks the reduced levels of depression are maintained indicating that experiencing the use of a behavioral health digital program with coaching is a sustainable approach that may create a positive environment leading to meaningful change.

We also hypothesized that breathing techniques may be an enhanced method to address and reduce the bodily and mental processes associated with anxiety. Anxiety causes sympathetic activation that is due to widespread depolarization throughout the brain and body [39]. Therefore, slow and deep breathing may lead to parasympathetic activation due to widespread inhibition and hyperpolarization [44]. It has been demonstrated that during respiration, inspiration inhibits sympathetic nervous system activity [82-84]. Furthermore, slow-deep breathing has been shown to cause near-complete sympathoinhibition [39,85]. Therefore, breathing techniques could be used as first-line and supplemental treatments for stress or anxiety and some emotional disorders [39].

Our findings demonstrate stronger anxiety reduction in the first 6 weeks of the digital intervention for users who more intensively used breathing exercises. In line with this, previous studies have shown that breathing exercises and yoga training for 6 weeks modulate sweating response to dynamic exercise and improve respiratory pressures [86]. Previous findings also indicated that a change in respiratory rate could be attributed to the effectiveness of the diaphragmatic breathing practice over the 8 weeks achieving mental health benefits such as reduced physiological stress [87].

Health behavior change theory posits that new health behaviors emerge when people achieve both knowledge and self-efficacy to implement the suggested knowledge [88,89]. We posit that the first 6 weeks of using specific activities such as breathing exercises through a digital platform, is a prime opportunity for reinforcing knowledge and building self-efficacy for the future. Moreover, the next 7-16 weeks remained with reduced levels of anxiety indicating sustainability and may imply behavioral change and adaptation of relaxation techniques. Coaching was not seen as critical with anxiety symptoms as with depressive symptoms. Based on these findings, one may speculate that how the interpersonal relationship interacts with treatment for anxiety needs to be further explored.

Recent reviews call for research that moves beyond examining aggregate mental health program effects to more nuanced investigations that explore the associations between the program building blocks and the clinical outcomes that disentangle the relative contributions of specific activity in digital health management protocols [52,55].

Our findings indicate that coaching and breathing exercises directly help people lower their depression and anxiety symptom levels respectively. These findings suggest that increasing digital interactions with specific activities in a lower engaged population may be an efficient way to optimize digital platforms supporting patients with depression and anxiety symptoms. New models of care and digital tools have the potential to help enable more people to access and benefit from high-quality care that is patient-centered, timely, equitable, and efficient. We expect that the analytical approach applied in this study will be beneficial for personalizing interventions and optimizing incentivization planning. This information could be used to further personalize outreach to encourage users to maintain their personal critical level of mental health activities. More research is required to further understand the current and potential moderating factors of digital intervention tools for mental health and how independent use of these tools or use with a coach influences the care of patients with depression and anxiety [90].

### Limitations

We note several limitations in this study. First, as in all studies involving retrospective real-world data, groups were not randomly assigned, and treatment protocols were not prescribed. Both factors create challenges for drawing causal conclusions. Surely, it is possible that people who chose to use digital therapeutic instruments were those who were the most motivated to make a change in their condition.

The dropout rate was 25.2% for the depression sample and 28.4% for the anxiety sample. These numbers come in line with the average dropout rate of mental health apps data, reported by a recent meta-analysis (26.2%) [91]. A significant challenge encountered in the use of mHealth interventions for patient care is attrition. Previous studies have highlighted that a substantial proportion, potentially up to 80%, of participants in mHealth interventions exhibit minimal engagement, which is defined as logging into the service fewer than twice. Moreover, only a small fraction of users consistently use the intervention over an extended period [92]. It is worth noting that while clinical trials frequently report retention rates of 70% or higher, these trials are often of short duration, some lasting less than 2 months. Therefore, these retention rates may not accurately reflect real-world use scenarios [93]. Dropout attrition is a threat to validity because it may introduce a selection bias [94]. Gender and age may affect dropout rates [93]. For this reason, we tested whether these factors are associated with the risk of a dropout using logistic regression. Neither age nor gender was related to the risk of dropout. Potentially, other sociodemographic factors that were not measured could be related to the risk of dropouts, limiting the validity and generalizability of our conclusions.

In this real-world data analysis, the time scale was designed to reflect weekly interval change over a 16-week period. However, the relationships of interest in this study could be potentially investigated in different scales emphasizing daily, or monthly outcomes. Owing to the difficulty in tracking daily changes in anxiety in real-world studies, most studies focus on weekly fluctuations. Another challenge regarding our data was that available demographic data were limited.

### Conclusions

Our findings suggest that the use of specific components in the mental health management platform as coaching sessions or breathing exercise, help users with depression and anxiety symptoms attend to and reduce symptoms of their mental health conditions. For users who participated in coaching sessions and breathing exercises, a significant decrease in depression and anxiety symptoms (respectively) was observed in the initial stages, reflecting the importance of session attendance for establishing the foundational rationale for future practice. Focusing on mental health support by using an app as an integrated part of the health self-management process significantly improved clinical outcomes. From a behavioral science perspective, directly focusing on actionable areas for improvement is expected to increase thought and action, and the amount of attention paid to actionable areas is likely to vary considerably among individuals. Future work investigating strategies for specific components that drive the focus and execution of actionable health behaviors in a highly personalized manner is needed. Furthermore, similar studies examining these, and other moderating factors that impact different behavioral health conditions including stress, anger, or substance use are warranted. Such a body of literature would help to move the field beyond the current state of “do digital tools work?” to a more nuanced understanding of what specific tools drive which specific clinical outcomes for which people under what circumstances.

## Abbreviations

DSM-IV: Diagnostic and Statistical Manual of Mental Disorders, 4th Edition
GAD-7: generalized anxiety disorder-7
HIPAA: Health Insurance Portability and Accountability Act
PHQ-9: patient health questionnaire-9
UP: Unified Protocol for Transdiagnostic Treatment Anxiety and Depressive Disorders

## References

[1] Friedrich MJ (2017). Depression is the leading cause of disability around the world. JAMA.

[2] COVID-19 Mental Disorders Collaborators (2021). Global prevalence and burden of depressive and anxiety disorders in 204 countries and territories in 2020 due to the COVID-19 pandemic. Lancet.

[3] GBD 2019 Mental Disorders Collaborators (2022). Global, regional, and national burden of 12 mental disorders in 204 countries and territories, 1990-2019: a systematic analysis for the Global Burden of Disease Study 2019. Lancet Psychiatry.

[4] Linde K, Sigterman K, Kriston L, Rücker G, Jamil S, Meissner K, Schneider A (2015). Effectiveness of psychological treatments for depressive disorders in primary care: systematic review and meta-analysis. Ann Fam Med.

[5] Plaisier I, Beekman ATF, de Graaf R, Smit JH, van Dyck R, Penninx BWJH (2010). Work functioning in persons with depressive and anxiety disorders: the role of specific psychopathological characteristics. J Affect Disord.

[6] Tan L, Wang MJ, Modini M, Joyce S, Mykletun A, Christensen H, Harvey SB (2014). Preventing the development of depression at work: a systematic review and meta-analysis of universal interventions in the workplace. BMC Med.

[7] (2022). Fit mind, fit job: from evidence to practice in mental health and work. OECD.

[8] Chisholm D, Sweeny K, Sheehan P, Rasmussen B, Smit F, Cuijpers P, Saxena S (2016). Scaling-up treatment of depression and anxiety: a global return on investment analysis. Lancet Psychiatry.

[9] Adler DA, McLaughlin TJ, Rogers WH, Chang H, Lapitsky L, Lerner D (2006). Job performance deficits due to depression. Am J Psychiatry.

[10] (2022). Psychological treatment for anxiety disorders—the elements for individualising treatment. NPS MedicineWise.

[11] Wuthrich VM, Meuldijk D, Jagiello T, Robles AG, Jones MP, Cuijpers P (2021). Efficacy and effectiveness of psychological interventions on co-occurring mood and anxiety disorders in older adults: a systematic review and meta-analysis. Int J Geriatr Psychiatry.

[12] National Collaborating Centre for Mental Health (UK) (2013). Social Anxiety Disorder: Recognition, Assessment and Treatment.

[13] Cuijpers P, Quero S, Dowrick C, Arroll B (2019). Psychological treatment of depression in primary care: recent developments. Curr Psychiatry Rep.

[14] Winter SE, Barber JP (2013). Should treatment for depression be based more on patient preference?. Patient Prefer Adherence.

[15] Lattie EG, Stiles-Shields C, Graham AK (2022). An overview of and recommendations for more accessible digital mental health services. Nat Rev Psychol.

[16] (2022). About the unified protocol. Unified Protocol Institute—Expert CBT Training.

[17] (2017). The promise of transdiagnostic treatments for anxiety disorders. Society of Clinical Psychology.

[18] Barlow DH, Farchione TJ, Fairholme CP, Ellard KK, Boisseau CL, Allen LB, Ehrenreich-May JT (2011). Unified Protocol for Transdiagnostic Treatment of Emotional Disorders: Therapist Guide.

[19] Ellard KK, Deckersbach T, Sylvia LG, Nierenberg AA, Barlow DH (2012). Transdiagnostic treatment of bipolar disorder and comorbid anxiety with the unified protocol: a clinical replication series. Behav Modif.

[20] Boswell JF, Anderson LM, Barlow DH (2014). An idiographic analysis of change processes in the unified transdiagnostic treatment of depression. J Consult Clin Psychol.

[21] Yan K, Yusufi MH, Nazari N (2022). Application of unified protocol as a transdiagnostic treatment for emotional disorders during COVID-19: an internet-delivered randomized controlled trial. World J Clin Cases.

[22] Carlucci L, Saggino A, Balsamo M (2021). On the efficacy of the unified protocol for transdiagnostic treatment of emotional disorders: a systematic review and meta-analysis. Clin Psychol Rev.

[23] Torous J, Cerrato P, Halamka J (2019). Targeting depressive symptoms with technology. Mhealth.

[24] Firth J, Torous J, Nicholas J, Carney R, Pratap A, Rosenbaum S, Sarris J (2017). The efficacy of smartphone-based mental health interventions for depressive symptoms: a meta-analysis of randomized controlled trials. World Psychiatry.

[25] Gan DZQ, McGillivray L, Han J, Christensen H, Torok M (2021). Effect of engagement with digital interventions on mental health outcomes: a systematic review and meta-analysis. Front Digit Health.

[26] Aboujaoude E, Salame W, Naim L (2015). Telemental health: a status update. World Psychiatry.

[27] Leong QY, Sridhar S, Blasiak A, Tadeo X, Yeo G, Remus A, Ho D (2022). Characteristics of mobile health platforms for depression and anxiety: content analysis through a systematic review of the literature and systematic search of two app stores. J Med Internet Res.

[28] Andersson G, Titov N, Dear BF, Rozental A, Carlbring P (2019). Internet-delivered psychological treatments: from innovation to implementation. World Psychiatry.

[29] Luo C, Sanger N, Singhal N, Pattrick K, Shams I, Shahid H, Hoang P, Schmidt J, Lee J, Haber S, Puckering M, Buchanan N, Lee P, Ng K, Sun S, Kheyson S, Chung DCY, Sanger S, Thabane L, Samaan Z (2020). A comparison of electronically-delivered and face to face cognitive behavioural therapies in depressive disorders: a systematic review and meta-analysis. EClinicalMedicine.

[30] Fitzpatrick KK, Darcy A, Vierhile M (2017). Delivering cognitive behavior therapy to young adults with symptoms of depression and anxiety using a fully automated conversational agent (Woebot): a randomized controlled trial. JMIR Ment Health.

[31] Grant AM (2003). The impact of life coaching on goal attainment, metacognition and mental health. Soc Behav Pers.

[32] Rosenberg BM, Kodish T, Cohen ZD, Gong-Guy E, Craske MG (2022). A novel peer-to-peer coaching program to support digital mental health: design and implementation. JMIR Ment Health.

[33] Wu MS, Chen SY, Wickham RE, O'Neil-Hart S, Chen C, Lungu A (2021). Outcomes of a blended care coaching program for clients presenting with moderate levels of anxiety and depression: pragmatic retrospective study. JMIR Ment Health.

[34] Mohr DC, Schueller SM, Tomasino KN, Kaiser SM, Alam N, Karr C, Vergara JL, Gray EL, Kwasny MJ, Lattie EG (2019). Comparison of the effects of coaching and receipt of app recommendations on depression, anxiety, and engagement in the intellicare platform: factorial randomized controlled trial. J Med Internet Res.

[35] Molloy A, Anderson PL (2021). Engagement with mobile health interventions for depression: a systematic review. Internet Interv.

[36] Eseadi C, Obidoa MA, Ogbuabor SE, Ikechukwu-Ilomuanya AB (2018). Effects of group-focused cognitive-behavioral coaching program on depressive symptoms in a sample of inmates in a Nigerian prison. Int J Offender Ther Comp Criminol.

[37] Meyer A, Wisniewski H, Torous J (2022). Coaching to support mental health apps: exploratory narrative review. JMIR Hum Factors.

[38] Hur JW, Kim B, Park D, Choi SW (2018). A scenario-based cognitive behavioral therapy mobile app to reduce dysfunctional beliefs in individuals with depression: a randomized controlled trial. Telemed J E Health.

[39] Jerath R, Crawford MW, Barnes VA, Harden K (2015). Self-regulation of breathing as a primary treatment for anxiety. Appl Psychophysiol Biofeedback.

[40] Naik GS, Gaur GS, Pal GK (2018). Effect of modified slow breathing exercise on perceived stress and basal cardiovascular parameters. Int J Yoga.

[41] Patin A, Hurlemann R (2011). Modulating amygdala responses to emotion: evidence from pharmacological fMRI. Neuropsychologia.

[42] Kim KH, Bang SW, Kim SR (2004). Emotion recognition system using short-term monitoring of physiological signals. Med Biol Eng Comput.

[43] Kop WJ, Synowski SJ, Newell ME, Schmidt LA, Waldstein SR, Fox NA (2011). Autonomic nervous system reactivity to positive and negative mood induction: the role of acute psychological responses and frontal electrocortical activity. Biol Psychol.

[44] Murik SE (2011). Polarization theory of motivations, emotions and attention. ArXiv. Preprint posted online on November 14 2011.

[45] Zope SA, Zope RA (2013). Sudarshan kriya yoga: breathing for health. Int J Yoga.

[46] Padival M, Quinette D, Rosenkranz JA (2013). Effects of repeated stress on excitatory drive of basal amygdala neurons in vivo. Neuropsychopharmacology.

[47] Leo AJ, Schuelke MJ, Hunt DM, Metzler JP, Miller JP, Areán PA, Armbrecht MA, Cheng AL (2022). A digital mental health intervention in an orthopedic setting for patients with symptoms of depression and/or anxiety: feasibility prospective cohort study. JMIR Form Res.

[48] Carswell K, Harper-Shehadeh M, Watts S, Van't Hof E, Abi Ramia J, Heim E, Wenger A, van Ommeren M (2018). Step-by-step: a new WHO digital mental health intervention for depression. Mhealth.

[49] Larsen ME, Nicholas J, Christensen H (2016). Quantifying app store dynamics: longitudinal tracking of mental health apps. JMIR Mhealth Uhealth.

[50] Sucala M, Cuijpers P, Muench F, Cardoș R, Soflau R, Dobrean A, Achimas-Cadariu P, David D (2017). Anxiety: there is an app for that. A systematic review of anxiety apps. Depress Anxiety.

[51] Ng MM, Firth J, Minen M, Torous J (2019). User engagement in mental health apps: a review of measurement, reporting and validity. Psychiatr Serv.

[52] Teachman BA, Drabick DAG, Hershenberg R, Vivian D, Wolfe BE, Goldfried MR (2012). Bridging the gap between clinical research and clinical practice: introduction to the special section. Psychotherapy (Chic).

[53] Lehtimaki S, Martic J, Wahl B, Foster KT, Schwalbe N (2021). Evidence on digital mental health interventions for adolescents and young people: systematic overview. JMIR Ment Health.

[54] Bry LJ, Chou T, Miguel E, Comer JS (2018). Consumer smartphone apps marketed for child and adolescent anxiety: a systematic review and content analysis. Behav Ther.

[55] Vis C, Kleiboer A, Prior R, Bønes E, Cavallo M, Clark SA, Dozeman E, Ebert D, Etzelmueller A, Favaretto G, Zabala AF, Kolstrup N, Mancin S, Mathiassen K, Myrbakk VN, Mol M, Jimenez JP, Power K, van Schaik A, Wright C, Zanalda E, Pederson CD, Smit J, Riper H, MasterMind consortium (2015). Implementing and up-scaling evidence-based eMental health in Europe: the study protocol for the MasterMind project. Internet Interv.

[56] Lungu A, Jun JJ, Azarmanesh O, Leykin Y, Chen CEJ (2020). Blended care-cognitive behavioral therapy for depression and anxiety in real-world settings: pragmatic retrospective study. J Med Internet Res.

[57] Fairburn CG, Patel V (2017). The impact of digital technology on psychological treatments and their dissemination. Behav Res Ther.

[58] Moshe I, Terhorst Y, Philippi P, Domhardt M, Cuijpers P, Cristea I, Pulkki-Råback L, Baumeister H, Sander LB (2021). Digital interventions for the treatment of depression: a meta-analytic review. Psychol Bull.

[59] Nwosu A, Boardman S, Husain MM, Doraiswamy PM (2022). Digital therapeutics for mental health: is attrition the Achilles heel?. Front Psychiatry.

[60] Barlow DH, Farchione TJ, Bullis JR, Gallagher MW, Murray-Latin H, Sauer-Zavala S, Bentley KH, Thompson-Hollands J, Conklin LR, Boswell JF, Ametaj A, Carl JR, Boettcher HT, Cassiello-Robbins C (2017). The unified protocol for transdiagnostic treatment of emotional disorders compared with diagnosis-specific protocols for anxiety disorders: a randomized clinical trial. JAMA Psychiatry.

[61] Nieuwsma JA, Trivedi RB, McDuffie J, Kronish I, Benjamin D, Williams JW (2012). Brief psychotherapy for depression: a systematic review and meta-analysis. Int J Psychiatry Med.

[62] Maeng D, Cornell AE, Nasra GS (2019). Utilization of an employee behavioral health program and its effects on outcomes for depression and anxiety disorders. J Occup Environ Med.

[63] Kunkle S, Yip M, Watson Ξ, Hunt J (2020). Evaluation of an on-demand mental health system for depression symptoms: retrospective observational study. J Med Internet Res.

[64] Farchione TJ, Fairholme CP, Ellard KK, Boisseau CL, Thompson-Hollands J, Carl JR, Gallagher MW, Barlow DH (2012). Unified protocol for transdiagnostic treatment of emotional disorders: a randomized controlled trial. Behav Ther.

[65] Chen TM, Huang FY, Chang C, Chung H (2006). Using the PHQ-9 for depression screening and treatment monitoring for Chinese Americans in primary care. Psychiatr Serv.

[66] (2023). PHQ-9 (Patient Health Questionnaire-9). MDCalc.

[67] Pinto-Meza A, Serrano-Blanco A, Peñarrubia MT, Blanco E, Haro JM (2005). Assessing depression in primary care with the PHQ-9: can it be carried out over the telephone?. J Gen Intern Med.

[68] Lee C, Round JM, Hanlon JG, Hyshka E, Dyck JRB, Eurich DT (2022). Generalized Anxiety Disorder 7-Item (GAD-7) scores in medically authorized cannabis patients-Ontario and Alberta, Canada. Can J Psychiatry.

[69] Spitzer RL, Kroenke K, Williams JBW, Löwe B (2006). A brief measure for assessing generalized anxiety disorder: the GAD-7. Arch Intern Med.

[70] (2020). Ethical and independent review services.

[71] Kohli N, Peralta Y, Zopluoglu C, Davison ML (2018). A note on estimating single-class piecewise mixed-effects models with unknown change points. Int J Behav Dev.

[72] Fundoiano-Hershcovitz Y, Hirsch A, Dar S, Feniger E, Goldstein P (2021). Role of digital engagement in diabetes care beyond measurement: retrospective cohort study. JMIR Diabetes.

[73] Fundoiano-Hershcovitz Y, Bacher D, Ritholz MD, Horwitz DL, Manejwala O, Goldstein P (2022). Blood pressure monitoring as a digital health tool for improving diabetes clinical outcomes: retrospective real-world study. J Med Internet Res.

[74] Fundoiano-Hershcovitz Y, Horwitz DL, Tawil C, Cohen O, Goldstein P (2022). The two-stage therapeutic effect of posture biofeedback training on back pain and the associated mechanism: a retrospective cohort study. Front Physiol.

[75] Kroenke K, Spitzer RL, Williams JBW, Löwe B (2010). The patient health questionnaire somatic, anxiety, and depressive symptom scales: a systematic review. Gen Hosp Psychiatry.

[76] Toussaint A, Hüsing P, Gumz A, Wingenfeld K, Härter M, Schramm E, Löwe B (2020). Sensitivity to change and minimal clinically important difference of the 7-item Generalized Anxiety Disorder Questionnaire (GAD-7). J Affect Disord.

[77] Hayes AM, Laurenceau JP, Feldman G, Strauss JL, Cardaciotto L (2007). Change is not always linear: the study of nonlinear and discontinuous patterns of change in psychotherapy. Clin Psychol Rev.

[78] Azelton KR, Crowley AP, Vence N, Underwood K, Morris G, Kelly J, Landry MJ (2021). Digital health coaching for type 2 diabetes: randomized controlled trial of healthy at home. Front Digit Health.

[79] Denneson LM, Trevino AY, Kenyon EA, Ono SS, Pfeiffer PN, Dobscha SK (2019). Health coaching to enhance psychological well-being among veterans with suicidal ideation: a pilot study. J Gen Intern Med.

[80] Silberman JM, Kaur M, Sletteland J, Venkatesan A (2020). Outcomes in a digital weight management intervention with one-on-one health coaching. PLoS One.

[81] Lambert MJ (2001). Psychotherapy outcome and quality improvement: introduction to the special section on patient-focused research. J Consult Clin Psychol.

[82] Dempsey JA, Sheel AW, St Croix CM, Morgan BJ (2002). Respiratory influences on sympathetic vasomotor outflow in humans. Respir Physiol Neurobiol.

[83] Czyzyk-Krzeska MF, Trzebski A (1990). Respiratory-related discharge pattern of sympathetic nerve activity in the spontaneously hypertensive rat. J Physiol.

[84] St Croix CM, Satoh M, Morgan BJ, Skatrud JB, Dempsey JA (1999). Role of respiratory motor output in within-breath modulation of muscle sympathetic nerve activity in humans. Circ Res.

[85] Seals DR, Suwarno NO, Dempsey JA (1990). Influence of lung volume on sympathetic nerve discharge in normal humans. Circ Res.

[86] Mahadevan SK, Balakrishnan S, Gopalakrishnan M, Prakash ES, Madanmohan (2008). Effect of six weeks yoga training on weight loss following step test, respiratory pressures, handgrip strength and handgrip endurance in young healthy subjects. Indian J Physiol Pharmacol.

[87] Ma X, Yue ZQ, Gong ZQ, Zhang H, Duan NY, Shi YT, Wei GX, Li YF (2017). The effect of diaphragmatic breathing on attention, negative affect and stress in healthy adults. Front Psychol.

[88] Lawson PJ, Flocke SA (2009). Teachable moments for health behavior change: a concept analysis. Patient Educ Couns.

[89] Lustria MLA, Noar SM, Cortese J, Van Stee SK, Glueckauf RL, Lee J (2013). A meta-analysis of web-delivered tailored health behavior change interventions. J Health Commun.

[90] Echelard JF (2021). Use of telemedicine in depression care by physicians: scoping review. JMIR Form Res.

[91] Torous J, Lipschitz J, Ng M, Firth J (2020). Dropout rates in clinical trials of smartphone apps for depressive symptoms: a systematic review and meta-analysis. J Affect Disord.

[92] Pfammatter AF, Mitsos A, Wang S, Hood SH, Spring B (2017). Evaluating and improving recruitment and retention in an mHealth clinical trial: an example of iterating methods during a trial. Mhealth.

[93] Wang Y, Xue H, Huang Y, Huang L, Zhang D (2017). A systematic review of application and effectiveness of mHealth interventions for obesity and diabetes treatment and self-management. Adv Nutr.

[94] Eysenbach G (2005). The law of attrition. J Med Internet Res.

